# Development and characterization of a monoclonal antibody blocking human TRPM4 channel

**DOI:** 10.1038/s41598-021-89935-5

**Published:** 2021-05-17

**Authors:** See Wee Low, Yahui Gao, Shunhui Wei, Bo Chen, Bernd Nilius, Ping Liao

**Affiliations:** 1 Calcium Signalling Laboratory, Department of Research, National Neuroscience Institute, 11 Jalan Tan Tock Seng, Singapore, 308433 Singapore; 2 Department of Cellular and Molecular Medicine, KU Leuven, Leuven, Belgium; 3 Duke-NUS Medical School, Singapore, Singapore; 4 Health and Social Sciences, Singapore Institute of Technology, Singapore, Singapore

**Keywords:** Neuroscience, Diseases, Medical research, Neurology

## Abstract

TRPM4 is a calcium-activated non-selective monovalent cation channel implicated in diseases such as stroke. Lack of potent and selective inhibitors remains a major challenge for studying TRPM4. Using a polypeptide from rat TRPM4, we have generated a polyclonal antibody M4P which could alleviate reperfusion injury in a rat model of stroke. Here, we aim to develop a monoclonal antibody that could block human TRPM4 channel. Two mouse monoclonal antibodies M4M and M4M1 were developed to target an extracellular epitope of human TRPM4. Immunohistochemistry and western blot were used to characterize the binding of these antibodies to human TRPM4. Potency of inhibition was compared using electrophysiological methods. We further evaluated the therapeutic potential on a rat model of middle cerebral artery occlusion. Both M4M and M4M1 could bind to human TRPM4 channel on the surface of live cells. Prolonged incubation with TRPM4 blocking antibody internalized surface TRPM4. Comparing to M4M1, M4M is more effective in blocking human TRPM4 channel. In human brain microvascular endothelial cells, M4M successfully inhibited TRPM4 current and ameliorated hypoxia-induced cell swelling. Using wild type rats, neither antibody demonstrated therapeutic potential on stroke. Human TRPM4 channel can be blocked by a monoclonal antibody M4M targeting a key antigenic sequence. For future clinical translation, the antibody needs to be humanized and a transgenic animal carrying human TRPM4 sequence is required for in vivo characterizing its therapeutic potential.

## Introduction

Transient receptor potential melastatin member 4 (TRPM4) is a voltage-dependent, non-selective monovalent cation channel^1^. Although TRPM4 is impermeable to Ca^2+^ ions, it can be activated by an elevated cytosolic Ca^2+^ level and modulated by ATP depletion. The voltage sensitivity of the channel is mediated by cytosolic Ca^2+^ level, temperature, phosphatidylinositol 4,5-bisphosphate content as well as calmodulin^2–4^. TRPM4 is expressed in many cell types and tissues, but TRPM4-deficient mice are viable and fertile with no obvious anatomical abnormalities^5^. TRPM4 plays a regulatory role in the functions of immune cells^1^, cardiovascular system^6^, nervous system^7,8^, and urinary bladder^9^. As TRPM4 conducts Na^+^ entry into the cell, its physiological role is likely via regulating membrane potential, particularly in excitable cells^10^.

TRPM4 has been studied extensively in human diseases. Mutations were identified in families with cardiac conduction problems including right bundle-branch block, tachycardia, and Brugada syndrome^6^. Aberrant TRPM4 expression has also been found in cancers from various tissues and organs such as prostate, liver, urinary bladder, cervix, colon, and large B cell^11^. However, the actual role of TRPM4 in cancer development remains largely unknown. In nervous system, TRPM4 contributes to the progression of diseases such as multiple sclerosis^12^, stroke^13^, head injury^14^, and spinal cord injury^15^. In these disorders, hypoxia is a common pathophysiological feature, always leading to higher intracellular Ca^2+^ levels and lower ATP concentration which can greatly enhance TRPM4 activity^16^. Upregulation of TRPM4 expression in together with an increased channel activity result in an excessive Na^+^ influx and are believed to cause oncotic cell death^15^.

Lack of specific and potent blockers remains a challenge in studying TRPM4. 9-phenanthrol which is commonly used in electrophysiology has been found to interact with TMEM16A channel and affect vascular contraction^17^. Additionally, 9-phenanthrol non-selectively inhibits transient outward, rapid delayed rectifier, and inward rectifier K^+^ currents in heart^18^, raising concerns about its specificity to TRPM4. Another TRPM4 blocker glibenclamide is widely used to control blood glucose level in patients with diabetes mellitus type 2. Glibenclamide inhibits K_ATP_ channels in pancreatic beta-cells via interacting with SUR1 subunit^19^. The inhibitory effect of glibenclamide on TRPM4 depends on the co-expression of SUR1 and TRPM4 at a certain ratio^20^. Without SUR1 co-expression, glibenclamide has no effect on TRPM4 activity^21^. Most recently, a small compound has been developed to block TRPM4 demonstrating both potency and specificity^22^. More experiments are needed to evaluate its in vivo functions.

During the past years, we have been working on inhibitors such as siRNA that can directly suppress TRPM4 activity^23^. Another approach is to use antibody to block TRPM4. A polyclonal antibody M4P was produced in our laboratory to target an antigenic epitope close to the channel pore^24^. M4P demonstrated specificity to TRPM4 and exhibited therapeutic potential in ameliorating reperfusion injury in a rat model of stroke^24^. As M4P is a polyclonal antibody which was designed against rodent TRPM4, it is unclear whether human TRPM4 channel can be blocked by an antibody, given the fact that human and rodent TRPM4 channels share a low homology. Here we describe the development of a monoclonal antibody M4M that can specifically block human TRPM4, and importantly, ameliorate hypoxia-induced oncosis.

## Material and methods

### Monoclonal antibody production and Western blot

A KLH conjugated 21-amino acid antigenic polypeptide (RDSDSNCSSEPGFWAHPPGAQ) which is close to the channel pore of human TRPM4 was used for antibody production. Biomatik, US was selected to generate the monoclonal antibody^25^. Based on ELISA results, two mouse monoclonal antibodies (subclass IgG1) M4M and M4M1 demonstrating strong binding affinity were selected for characterization. Antibodies were stored at a concentration of 1 mg/mL at − 80 °C.

Western blot has been described in our previous publications^23,24,26,27^. In brief, HEK 293 cells grown in 6-well petri dishes were transfected with 4 μg human or mouse TRPM4 plasmid using lipofectamine 2000 transfection reagent (Cat#11668019, Thermo Fisher Scientific, MA, USA). 24 h after transfection, 80 μg of total protein was resolved on 10% SDS-PAGE gels at 80 V, and electrophoretically transferred to PVDF membranes (1620177, Bio-Rad, CA, USA) at 100 V for 2 h at 4 °C. After blocking with StartingBlock (PBS) blocking buffer (37538, Thermo Fisher Scientific, MA, USA) for 1 h at room temperature, the membranes were incubated overnight at 4 °C with primary antibodies: M4M1 (1:300), M4M (1:300), M4P (1:300) and anti-actin (A1978, Sigma-Aldrich, MI, USA, 1:1000). After washing away primary antibodies, the membranes were incubated with secondary antibodies against mouse or rabbit IgG for 1 h at room temperature. Primary and secondary antibodies were prepared in StartingBlock (PBS) blocking buffer with 0.05% Tween20 (P7949, Sigma-Aldrich, MI, USA). Washing buffers contained 0.1% Tween20 dissolved in phosphate-buffered saline (PBS). Amersham ECL Western Blotting Analysis System (RPN2109, GE Healthcare, IL, USA) was used and the bands were visualized using a medical X-ray processor (MXP-2000, Kodak, NY, USA). Quantification was done using ImageJ.

### Surface protein biotinylation

TRPM4 surface expression was characterized using an EZLink Sulfo-NHS-Biotinylation Kit (Thermo Fisher Scientific, MA, USA). The details have been described previously^28^ with slight modification. HEK 293 cells transfected with human TRPM4 (Myc-DDK-tagged) were incubated with monoclonal M4M1 or monoclonal M4M at a concentration of 1.3 μg/mL for 6 h. The cells were then treated with 0.25 mg/mL Biotin and shaken for 1 h at 4 °C. Unbound biotin was removed by incubation with quenching buffer for 20 min and washed with cold tris-buffered saline (TBS). Protein concentrations of cell lysates were measured with Pierce BCA Protein Assay Kit (23227, Thermo Fisher Scientific, MA, USA). 10 µL cell lysates was kept for SDS-PAGE analysis. The remaining cell lysates was incubated with NeutrAvidin (Thermo Fisher Scientific, MA, USA) overnight at 4 °C to pull down the biotinylated surface proteins. The precipitates were boiled in 2 × loading buffer to elute Avidin-bound for SDS-PAGE analysis and subsequent western blot. For the expression of surface protein, anti-Transferrin Receptor (TfR, 13-6800, Thermo Fisher Scientific, MA, USA, 1:1000) was used as a loading control, whereas anti-actin was used as a control for cytosolic protein.

### Immunofluorescent staining

HEK 293 cells expressing human TRPM4 were seeded on Poly-D-lysine coated coverslips in 12 wells plate. 24 h post transfection, M4M, M4M1 or control mouse IgG (subclass IgG1) was added into the culture medium to a concentration of 0.4 µg/mL. After incubation for 30 min, 3 h, or 6 h, the cells were fixed with 4% paraformaldehyde, followed by washing three times with 0.2% Triton X-100 phosphate-buffered saline (PBST). The samples were then blocked by 10% fetal bovine serum in 0.2% PBST for 1 h. Secondary antibody against mouse IgG is conjugated with Alexa Fluor 594 which was purchased from Thermo Fisher Scientific, MA, USA. To validate whether the Myc-DDK-tagged human TRPM4 was successfully transfected into HEK 293 cells, anti-c-myc (C3956, Sigma-Aldrich, MI, USA, 5 µg/mL) was used to stain the c-myc positive cells. All slides were finally mounted with FluorSave reagent (345789, Millipore, MA, USA), and visualized by a confocal microscope (Fluoview BX61, Olympus, Tokyo, Japan). We used ImageJ to quantify the fluorescent intensity. Cytosolic and membrane staining patterns were analyzed using a method described previously^29^. Images with fluorescent staining that occupied 30% of the cytosol and above were determined as cytosolic staining. The number of cells with cytosolic staining over the total number of cells was calculated to give a ratio of cytosolic staining. For surface immunostaining, M4M, M4M1 or control mouse IgG at a concentration of 0.4 µg/mL were added to the HEK 293 cells expressing human TRPM4. After incubation for 1 h, the cells were fixed with 4% paraformaldehyde. Following similar treatment, the cells were incubated with secondary antibodies against mouse IgG conjugated with Alexa Fluor 594 (Thermo Fisher Scientific, MA, USA). Wheat Germ Agglutinin (WGA) Alexa Fluor 488 (W11261, Thermo Fisher Scientific, MA, USA 5 µg/mL) was used to stain plasma membrane and DAPI (D9542, Sigma-Aldrich, MI, USA, 1 µg/mL) was used to stain nuclei. For TRPM4 co-stained with lysosome marker LAMP1, TRPM4 was labeled with anti-C-Myc (C3956, Sigma-Aldrich, MI, USA, 5 µg/mL), and LAMP1 was labeled with anti-LAMP1 (AB13523, Abcam, MA, USA, 5 µg/mL). Secondary antibodies against rabbit IgG (conjugated with Alexa Fluor 488) and mouse IgG (conjugated with Alexa Fluor 594) were purchased from Thermo Fisher Scientific, MA, USA. DAPI (D9542, Sigma-Aldrich, MI, USA, 1 µg/mL) was used to stain nuclei.

For human brain immunostaining, the tissue was obtained from a 76-year-old female patient who died from hemorrhagic stroke. Cryosection was performed at 10 µm in thickness. Following fixation with 4% paraformaldehyde, the brain slice was incubated in 100 μL blocking serum (10% fetal bovine serum in 0.2% PBST) for 1 h, and subsequently with primary antibodies. Primary antibodies include M4M (5 μL/mL), anti-NeuN (ABN78, Merck, USA, 1:400), and anti-vWF (AB7356, Merck, USA, 1:200). The staining and image taking process are similar to those for HEK 293 cells.

### Electrophysiology

Whole-cell patch clamp was used to characterize the electrophysiological properties of M4M and M4M1 antibodies as reported previously^24^. In brief, HEK293 cells were transfected with 2 μg human TRPM4 (Myc-DDK-tagged) using lipofectamine 2000 transfection reagent (Cat#11668019, Thermo Fisher Scientific, MA, USA). 24 h after transfection, whole-cell currents were recorded at room temperature using a patch clamp amplifier (Multiclamp 700B equipped with Digidata 1440A, Molecular Devices, CA, USA). Patch electrodes were pulled using a Flaming/Brown micropipette puller (P-1000, Sutter Instrument, CA, USA) and polished with a microforge (MF200, World Precision Instruments Inc. FL, USA). The bath solution contained (in millimole/litre) NaCl 140, CaCl_2_ 2, KCl 2, MgCl_2_ 1, glucose 20, and HEPES 20 at pH 7.4. The internal solution contained (in millimole/litre) CsCl 156, MgCl_2_ 1, EGTA 10, and HEPES 10 at pH 7.2 adjusted with CsOH. Additional Ca^2+^ was added in the pipette solution to get 7.4 μM free Ca^2+^, calculated using WEBMAXC v2.10. Monoclonal antibodies M4M, M4M1 or mouse control IgG (subclass IgG1) was added into bath solution 30 min before recording at a concentration of 20.8 μg/mL. The current–voltage relations were measured by applying voltage ramps for 250 ms from − 80 to + 80 mV at a holding potential of 0 mV. The sampling rate was 20 kHz and the filter setting was 1 kHz. Data were analysed using pClamp10, version10.2 (Molecular Devices, CA, USA).

For human brain microvascular endothelial cells, hypoxia was induced by applying a bath solution containing 5 mM NaN_3_ and 10 mM 2-deoxyglucose (2-DG) continuously through a MicroFil (34 Gauge, World Precision Instruments Inc. USA) around 10 μm away from the recording cells. The flow rate was set at 200 μL/min. The bath solution and internal solution are the same as those used for HEK 293 cells. The ramp protocols are also similar, except for the holding potential to be set at − 70 mV or a depolarized 0 mV representing hypoxia and ramps were 250 ms from − 100 to + 100 mV.

### Animal model and study design

This study was approved and conducted in accordance with the guidelines of the Institutional Animal Care and Use Committee of the National Neuroscience Institute, Singapore. All experiments were performed according to Stroke Therapy Academic Industry Roundtable (STAIR) recommendations^30^ and ARRIVE guidelines (https://arriveguidelines.org). The animals were housed with temperature maintained at around 23 °C and 12/12-h light/dark cycle was set. Pelleted food and water were available for the animals. The animals were monitored on a daily basis. All researchers involved in the study were blinded to the intervention. Allocation of animal treatment was randomised by throwing a dice. Transient middle cerebral artery occlusion (MCAO) method has been described previously^26^. In brief, male Wistar rats (250–280 g of body weight) were anesthetised with ketamine (75 mg/kg) and xylazine (10 mg/kg) intraperitoneally. A Laser-Doppler flowmetry (moorVMS-LDF2, Moor Instruments Inc., DE, USA) was used to measure cerebral blood flow. Animals with less than 70% cerebral blood flow reduction were excluded from the study. Heart rate, blood pressure, and rectal temperature were monitored using a data acquisition system PowerLab 4/35 from AD Instruments. The body temperature was maintained at 37 °C ± 0.5 °C with a warm pad throughout the operation. The left common carotid artery (CCA), internal carotid artery (ICA), and external carotid artery (ECA) were dissected out. A silicon-coated filament (0.37 mm, Cat #403756PK10, Doccol Corp, CA, USA) was inserted into the left ICA through ECA. A single dose of 100 μg of antibody was injected intravenously via tail vein at 2 h after occlusion (1 h before recanalization). Reperfusion was achieved by removing the filament gently from the ECA at 3 h following occlusion.

### Infarct volume measurement and motor functional assessment

2,3,5-Triphenyltetrazolium chloride (TTC) staining is a convenient procedure for detection of brain infarcts in stroke. 24 h after surgery, the brains were collected. After removing the cerebellum and overlying membranes, the brains were sectioned into 8 slices with 2 mm in thickness. The slices were then incubated in a 0.1% solution of TTC (T4375, Sigma-Aldrich, MI, USA) for 30 min at 37 °C. After the slices were scanned and analysed using an image analyser system (HP Scanjet G3110, HP Inc, CA, USA), infarct volume was calculated with a correction of oedema as described previously^26^.

The motor functions in animals after surgery were evaluated using a Rotarod produced by Ugo Basile, Gemonio, Italy. Before operation, all rats received 3 training trials with 15-min intervals for 5 consecutive days. The rotarod was set to accelerate from 4 to 80 rpm within 10 min. The mean duration of time that the animals remained on the device 1 day before operation was recorded and set as baseline control. One day following surgery, the mean duration of latency was recorded and compared with baseline. Animals with the performance higher than baseline were excluded from the analysis.

### Statistical analysis

Data are expressed as the mean ± s.e.m. Statistical analyses were performed using GraphPad Prism version 6.0. Paired student’s t test was used to compare two means. One-way ANOVA was used to compare 3 means or more with Bonferroni’s post hoc analysis. Two-way ANOVA with Bonferroni’s multiple comparison test was used to analyze multiple groups with various time points. *P* < 0.05 was considered significant.

## Results

### Generation and characterization of monoclonal antibodies against human TRPM4

**Figure 1:**
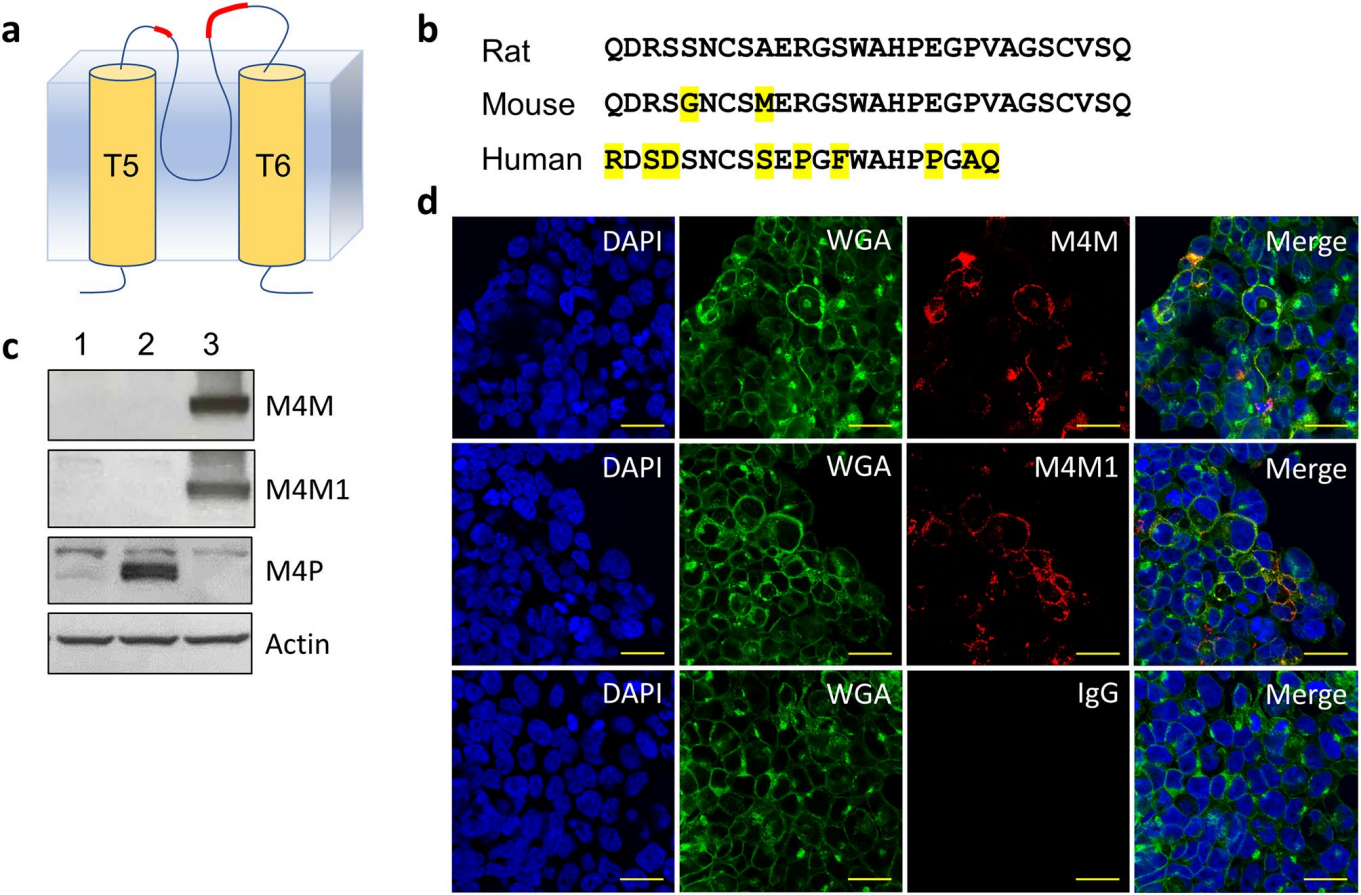
Generation of monoclonal antibody against human TRPM4 channel. (**a**) The extracellular antigenic epitope RDSDSNCSSEPGFWAHPPGAQ (labelled in red) is located close to the human TRPM4 channel pore. (**b**) Alignment of the antigenic sequences from rat and human TRPM4 channels for generating polyclonal antibody M4P against rat TRPM4 and monoclonal antibody against human TRPM4. Mouse sequence was also demonstrated for comparison. Residues different from rat sequence were highlighted in yellow. (**c**) Western blot on HEK 293 cells transfected with pcDNA c-Myc empty vector (lane 1), mouse TRPM4 (lane 2) and human TRPM4 (lane 3). M4M and M4M1 are two mouse monoclonal antibodies raised against human TRPM4. M4P is a polyclonal antibody raised against rodent TRPM4. Full-length blots are presented in Supplementary Fig. S1. (**d**) Live human TRPM4 expressing HEK 293 cells were incubated with M4M, M4M1 or control mouse IgG at a concentration of 0.4 µg/mL for 1 h, followed by fixation and staining with secondary antibody against mouse IgG. Cell membrane was labeled with Wheat Germ Agglutinin (WGA). Scale bars: 20 µm.

Based on hydrophobicity analysis, we selected an antigenic epitope which is between transmembrane segments 5 (T5) and 6 (T6) and close to the channel pore of human TRPM4 channel (Fig. 1a) for raising the blocking monoclonal antibody. This epitope is similar to what we targeted for generating the polyclonal antibody M4P against rat TRPM4^24^. This 21-amino acid antigenic polypeptide from human TRPM4 is shorter than the 28-amino acid sequence for M4P production (Fig. 1b). Of the 21 amino acids, 4 are part of the linker between T5 and the pore loop. The remaining 17 amino acids is part of the linker between the pore loop and T6. The 21-amino acid polypeptide is 57.1% (12/21) homologous to the rat TRPM4 sequence. For comparison, the mouse sequence is 93% homologous (26/28) to the rat sequence.

Two lines of monoclonal antibody M4M and M4M1 have been produced with a potential for binding to human TRPM4. Western blot was first performed on HEK 293 cells being transfected with human or mouse TRPM4 channels. Both M4M and M4M1 successfully detected human TRPM4, whereas neither antibody could identify mouse TRPM4 (Fig. 1c) because of a low homology between mouse and human TRPM4 channels (Fig. 1b). In contrast, M4P antibody, which was designed for rat TRPM4^24^, showed specificity for mouse TRPM4 due to a high homology between rat and mouse TRPM4 channels (93%). Accordingly, M4P did not cross react with human TRPM4 (Fig. 1c). Next, we examined whether M4M or M4M1 could bind to TRPM4 in live cells. Live HEK 293 cells expressing human TRPM4 were incubated with M4M or M4M1 for 1 h and subsequently fixed and stained with secondary antibodies against mouse IgG. Both M4M and M4M1 were found on the surface of HEK 293 cells, colocalizing with cell membrane marker Wheat Germ Agglutinin (WGA), whereas control mouse IgG did not stain the cells (Fig. 1d).

**Figure 2:**
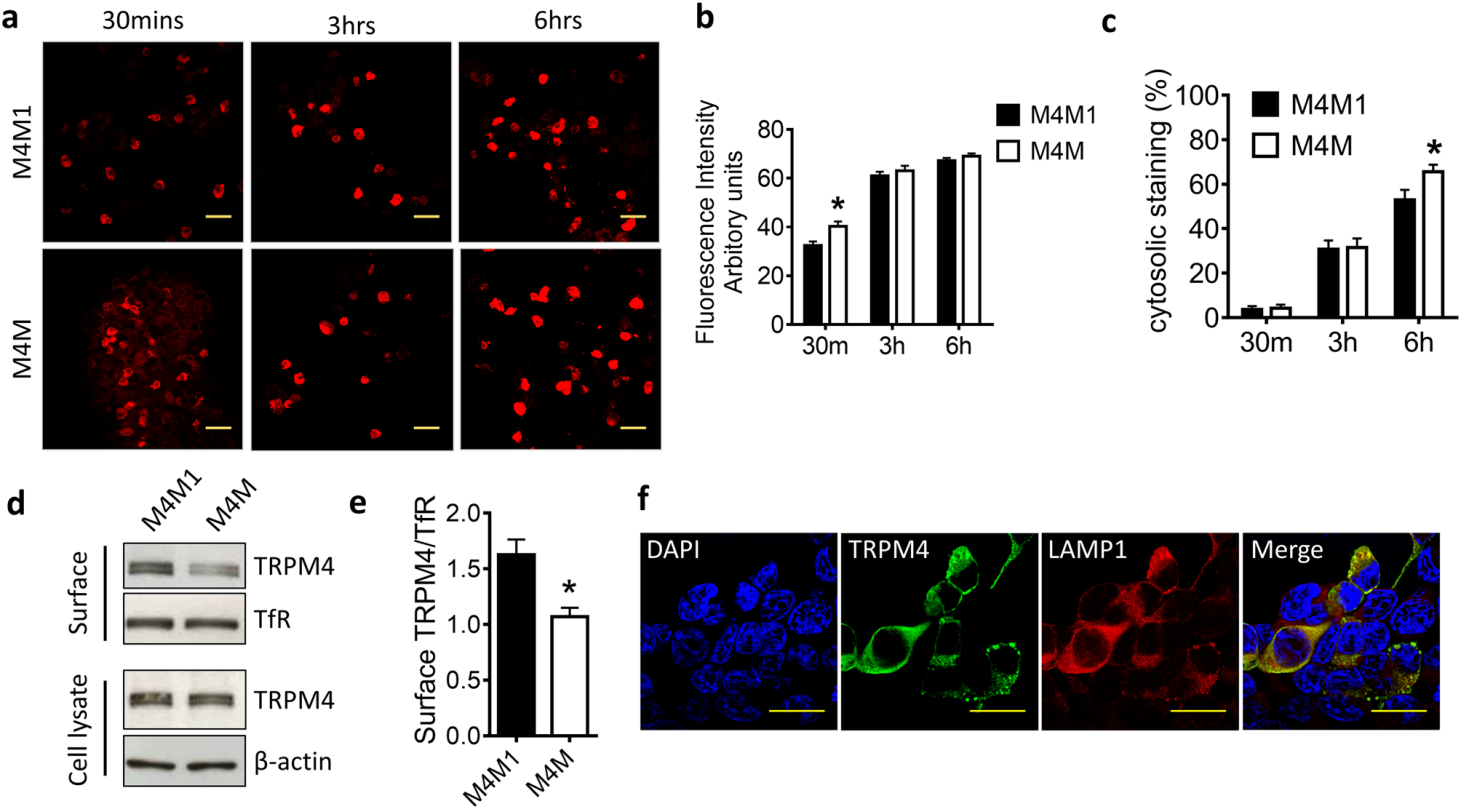
Detection of surface human TRPM4 using M4M and M4M1. (**a**) Live human TRPM4 expressing HEK 293 cells were incubated with M4M or M4M1 at a concentration of 0.4 µg/mL for 30 min, 3 h or 6 h, followed by fixation and staining with a secondary antibody against mouse IgG. Scale bars: 20 µm. (**b**) Summary of the fluorescent intensity. n = 4 experiments. (**c**) Summary of time dependent cytosolic staining. n = 4 experiments. (**d**) Detection of surface biotinylated human TRPM4 protein. Full-length blots are presented in Supplementary Fig. S2. (**e**) Summary of surface TRPM4 quantification. n = 4 experiments. (**f**) Colocalization of TRPM4 with lysosome marker LAMP1. Live human TRPM4 expressing HEK 293 cells were incubated with M4M for 24 h. Scale bars: 20 µm. In (**b,c**), statistical analysis was performed by two-way ANOVA with Bonferroni post-hoc analysis; in (**e**) by paired *t* test. *p < 0.05.

Prolonged antibody-channel binding has been shown to internalize surface channel expression^24^. To examine whether M4M or M4M1 has similar effect, both antibodies were added onto live cells and incubated for 30 min, 3 h, and 6 h. Secondary antibody against mouse IgG was used to detect the staining patterns of M4M or M4M1 (Fig. 2a). All procedures were performed in parallel under the same conditions including the exposure time for image taking. Quantification of fluorescent intensity showed that M4M had a higher intensity than M4M1 at 30 min after antibody incubation (Fig. 2b), whereas no difference was detected at 3 h or 6 h. Next, we examined the patterns of immunostaining: surface and cytosolic. Software analysis showed that at 30 min or 3 h, both antibodies demonstrated a similar pattern of immunostaining. Whereas at 6 h, more cytosolic staining was identified in the cells treated with M4M (Fig. 2c). Further surface biotinylation assay was performed to compare the ability between M4M and M4M1 to downregulate surface TRPM4 expression (Fig. 2d). After 6-h incubation, expression of total TRPM4 protein was not changed in both groups. Again, M4M showed more potency in reducing surface TRPM4 expression (Fig. 2d,e). As prolonged incubation of M4M increases intracellular staining, we examined TRPM4 subcellular expression (Fig. 2f). 24 h after M4M incubation, intracellular TRPM4 was found colocalized with lysosome marker LAMP1.

### M4M blocks human TRPM4 currents and ameliorates hypoxia-induced cell swelling

**Figure 3:**
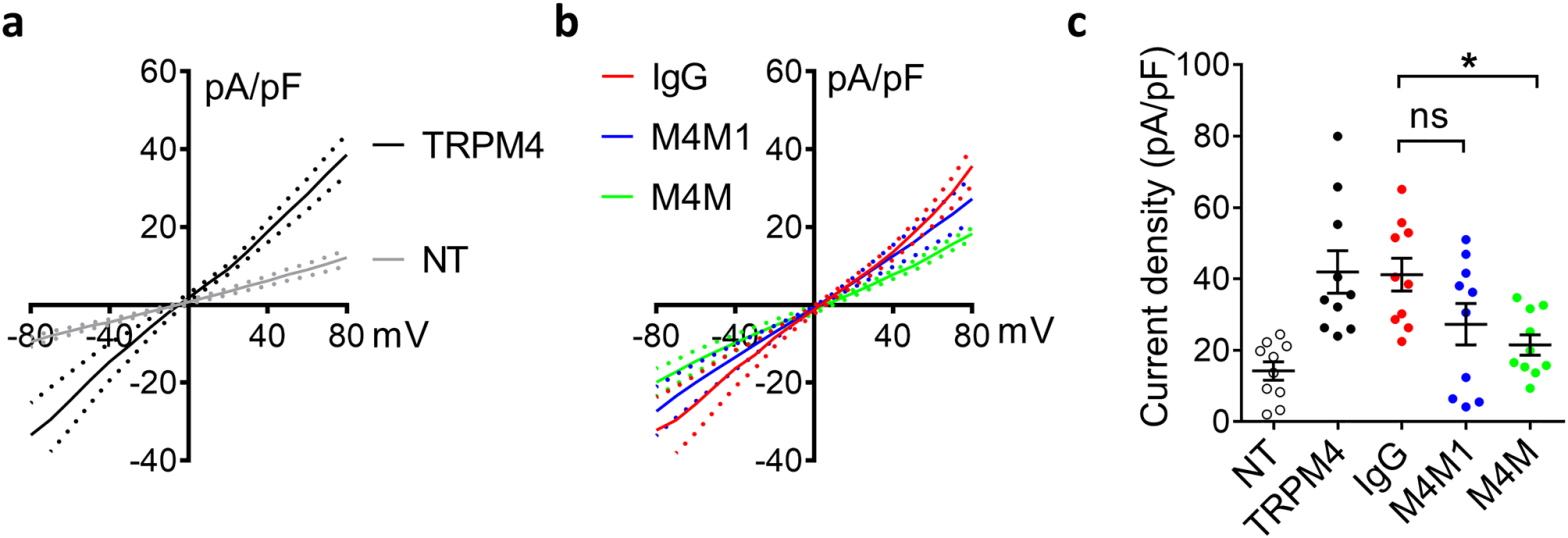
M4M blocks human TRPM4 currents. (**a**) The current voltage relationships of TRPM4 were obtained from HEK 293 cells transfected with human TRPM4 and compared to control HEK 293 cells without transfection (NT). Ramp protocols were applied from − 80 to + 80 mV with a holding potential at 0 mV. The data were presented as means ± s.e.m. (**b**) Comparison of human TRPM4 currents after the treatments of control IgG, M4M1 and M4M. Human TRPM4-transfected HEK 293 cells were incubated with control mouse IgG, M4M or M4M1 at a concentration of 20.8 μg/ml for 30 min before patch clamping. (**c**) Summary of current densities at + 80 mV. For (**a**–**c**), n = 10 cells for each group. *p < 0.05, *ns* no significance, One-way ANOVA with Bonferroni’s post hoc analysis.

To examine whether M4M or M4M1 could block human TRPM4 currents, we first transfected HEK 293 cells with human TRPM4 plasmids. The current voltage relationships reveal that both inward and outward currents were significantly increased in cells expressing TRPM4 (Fig. 3a). Next, human TRPM4-transfected HEK 293 cells were incubated with control mouse IgG, M4M or M4M1 at a concentration of 20.8 μg/mL for 30 min before TRPM4 currents were recorded using a ramp protocol (Fig. 3b). Analysis on currents at + 80 mV showed that M4M treatment could significantly reduce TRPM4 currents comparing to IgG treatment or TRPM4 transfection alone (Fig. 3c). It should be noted that although M4M1 treatment demonstrates a trend of current reduction, there is no statistical difference from IgG treatment or TRPM4 transfection alone (Fig. 3c).

**Figure 4:**
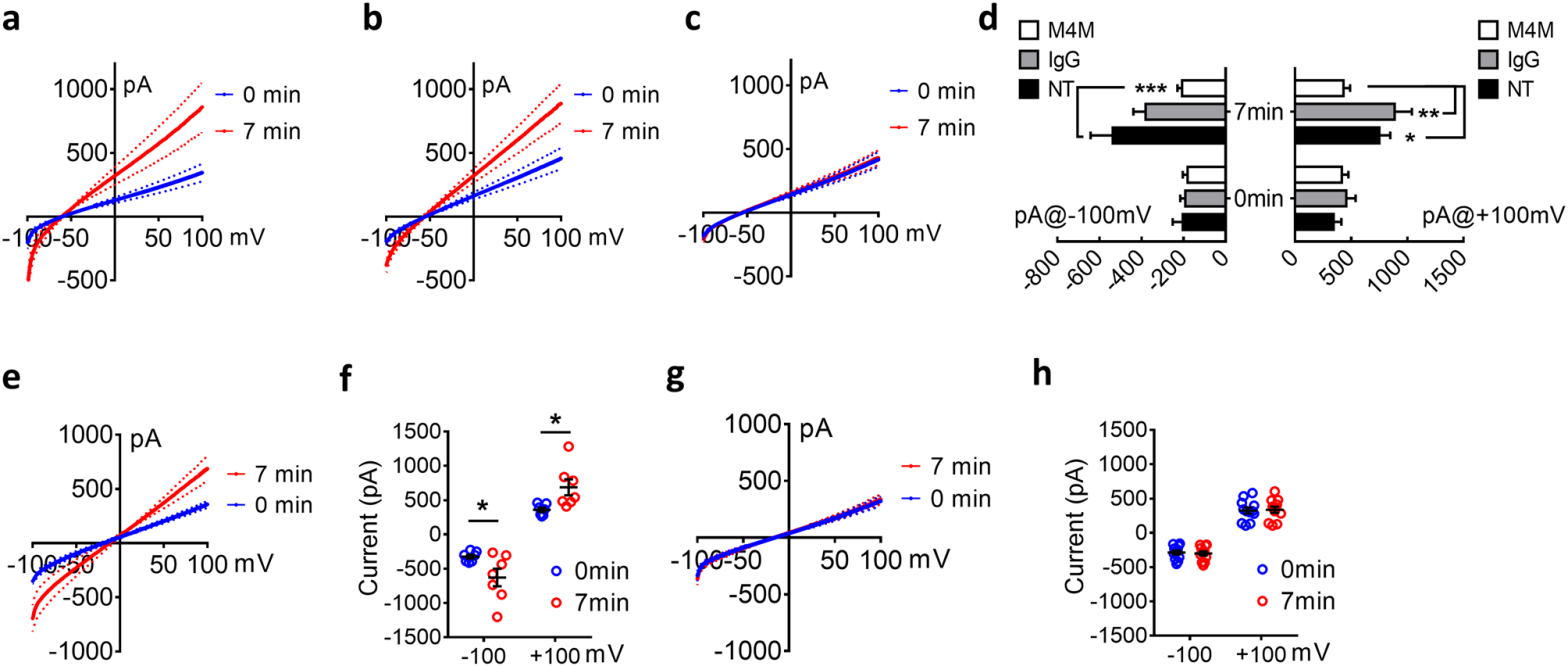
M4M blocks TRPM4 currents in cultured Human Brain Microvascular Endothelial Cells (HBMECs). (**a**) The current voltage relationships of TRPM4 from HBMECs before and after 7-min hypoxic induction. 250-ms voltage ramps from − 100 to + 100 mV was applied at 1 min interval with a holding potential of − 50 mV. Hypoxia was induced by adding 5 mM NaN3 and 10 mM 2-DG to the cells for 7 min. The data were presented as means ± s.e.m. (**b**) The current voltage relationships of TRPM4 from HBMECs pre-treated with control mouse IgG at a concentration of 20.8 μg/mL for 30 min. (**c**) The current voltage relationships of TRPM4 from HBMECs pre-treated with M4M at a concentration of 20.8 μg/mL for 30 min. (**d**) Summary of currents at − 100 mV and + 100 mV at 0 min before hypoxic induction and 7 min after hypoxic induction. For (**a**–**d**), Non-transfected group (NT): n = 11 cells; Control mouse IgG: n = 7 cells; M4M: n = 11 cells. (**e**) The current voltage relationships of HBMECs pre-treated with control mouse IgG with 0 mV holding potential. (**f**) Summary of currents at − 100 mV and + 100 mV at 0 min and 7 min. n = 7 cells. (**g**) The current voltage relationships of HBMECs pre-treated with M4M at 0 mV holding potential. (**h**) Summary of currents at − 100 mV and + 100 mV at 0 and 7 min. n = 12 cells. Statistical analysis in (**d**,**f**,**h**) were performed using Two-way ANOVA with Bonferroni’s post hoc test. *p < 0.05, **p < 0.01, ***p < 0.001.

As M4M exhibited a better blocking effect on human TRPM4 channel, we next focused on the electrophysiological properties of M4M on cultured human brain microvascular endothelial cells (HBMECs), particularly under hypoxia. HBMECs were first held at − 50 mV which is close to the resting membrane potential^31^. Acute hypoxia was achieved by adding 5 mM NaN3 and 10 mM 2-DG to the cells for 7 min. In line with a previous study showing that hypoxia activates TRPM4 activity^15^, hypoxia greatly enhanced TRPM4 currents in non-treated HBMECs (Fig. 4a). When HBMECs were pre-incubated with control mouse IgG, hypoxia again activated TRPM4 activity (Fig. 4b). In sharp contrast, M4M treatment completely inhibited the current increase under hypoxia (Fig. 4c). As summarized in Fig. 4d, all three groups (non-treated, control IgG, and M4M) showed similar levels of current at baseline 0 min. At 7 min, both control non-treated group and control IgG group exhibited enhanced current, indicating the activation of TRPM4 channel under hypoxia condition. There is no difference between non-treated group and control IgG group, suggesting that control IgG does not inhibit TRPM4 current. Whereas, the inhibitory effect of M4M is prominent under hypoxia treatment at 7 min (Fig. 4d).

As − 50 mV is a resting membrane potential of healthy endothelial cells^32^, we further held membrane potential at depolarized 0 mV and study the effect of M4M. At this depolarized membrane potential, hypoxia activated TRPM4 channel in IgG treated cells (Fig. 4e,f). Again, M4M treatment significantly inhibited TRPM4 current increase under hypoxia (Fig. 4g,h).

**Figure 5:**
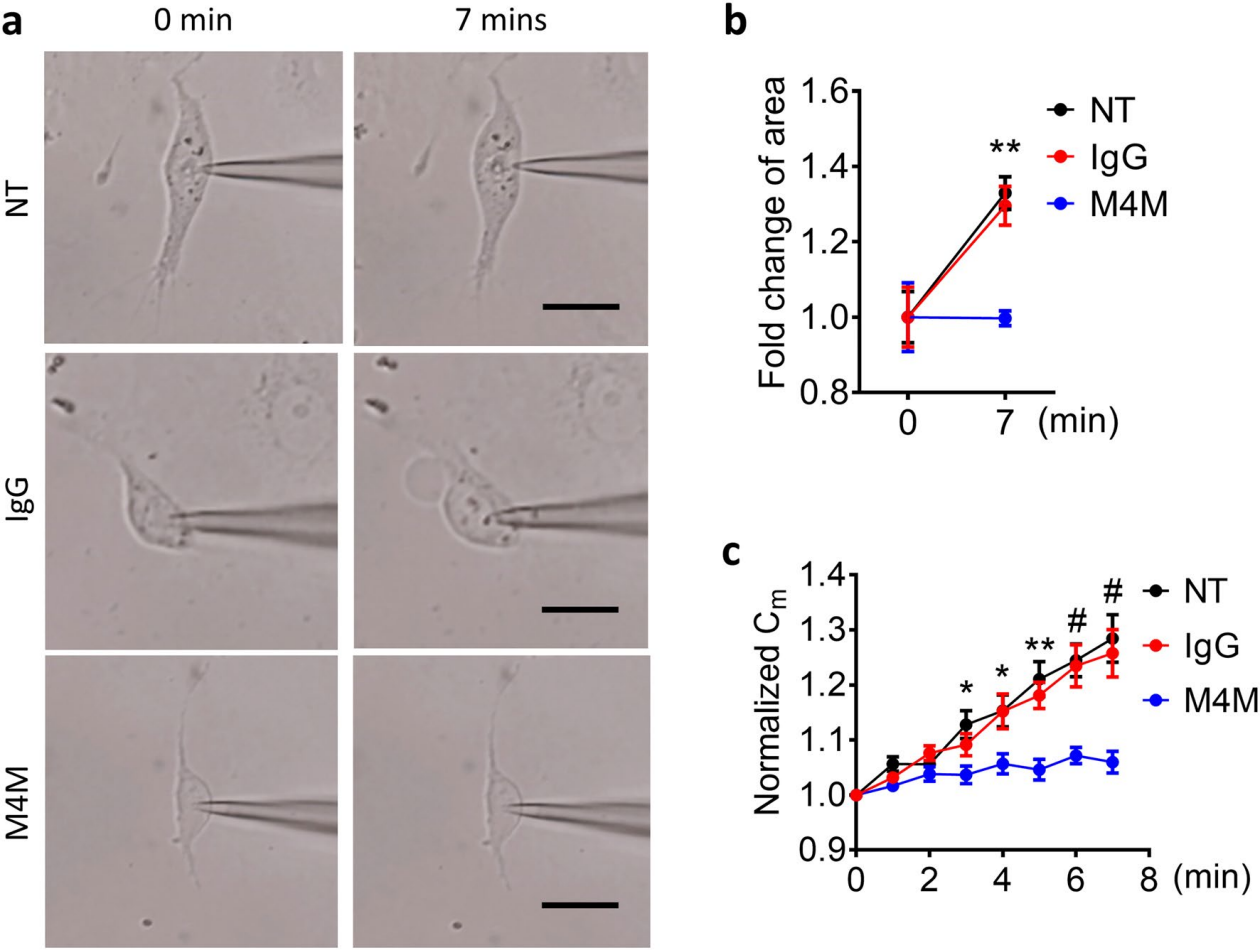
M4M prevents hypoxia-induced cell swelling in HBMECs. (**a**) Sample images of HBMECs before and after 7-min hypoxia treatment. HBMECs were pre-treated with control mouse IgG or M4M at a concentration of 20.8 μg/mL for 30 min. NT: non-treated. Scale bars: 10 µm. (**b**) Summary of cell areas. n = 8 for IgG and n = 7 for NT and M4M. (**c**) Time course of normalized membrane capacitance (Cm) under 7-min hypoxic induction. n = 9 cells for NT; Control mouse IgG: n = 8 cells; M4M: n = 9 cells. Statistical analysis in (**b,c**) were performed using Two-way ANOVA with Bonferroni’s post hoc test. *p < 0.05, **p < 0.01, ^#^p < 0.0001 for M4M vs IgG or NT.

We have reported previously that polyclonal antibody M4P could ameliorate hypoxia-mediated cell swelling^24^. Here, M4M was examined on HBMECs using similar protocols. We first took images of HBMECs at baseline 0 min and at 7 min after hypoxia treatment (Fig. 5a). Image quantification indicated that hypoxia significantly increased the cell area in IgG treated and in non-treated group (Fig. 5b). In sharp contrast, no change in cell area was observed in M4M treated cells. We further calculated the change of cell membrane capacitance (Cm) which is an indicator of cell volume at 1 min interval during a 7-min hypoxia induction. Cm was increased gradually during the hypoxia treatment in both non-treated and IgG treated groups. Conversely, the Cm remained unchanged in M4M treated cells (Fig. 5c). These data collectively indicate that M4M could suppress hypoxia-induced cell swelling.

### Effect of M4M in stroke

**Figure 6:**
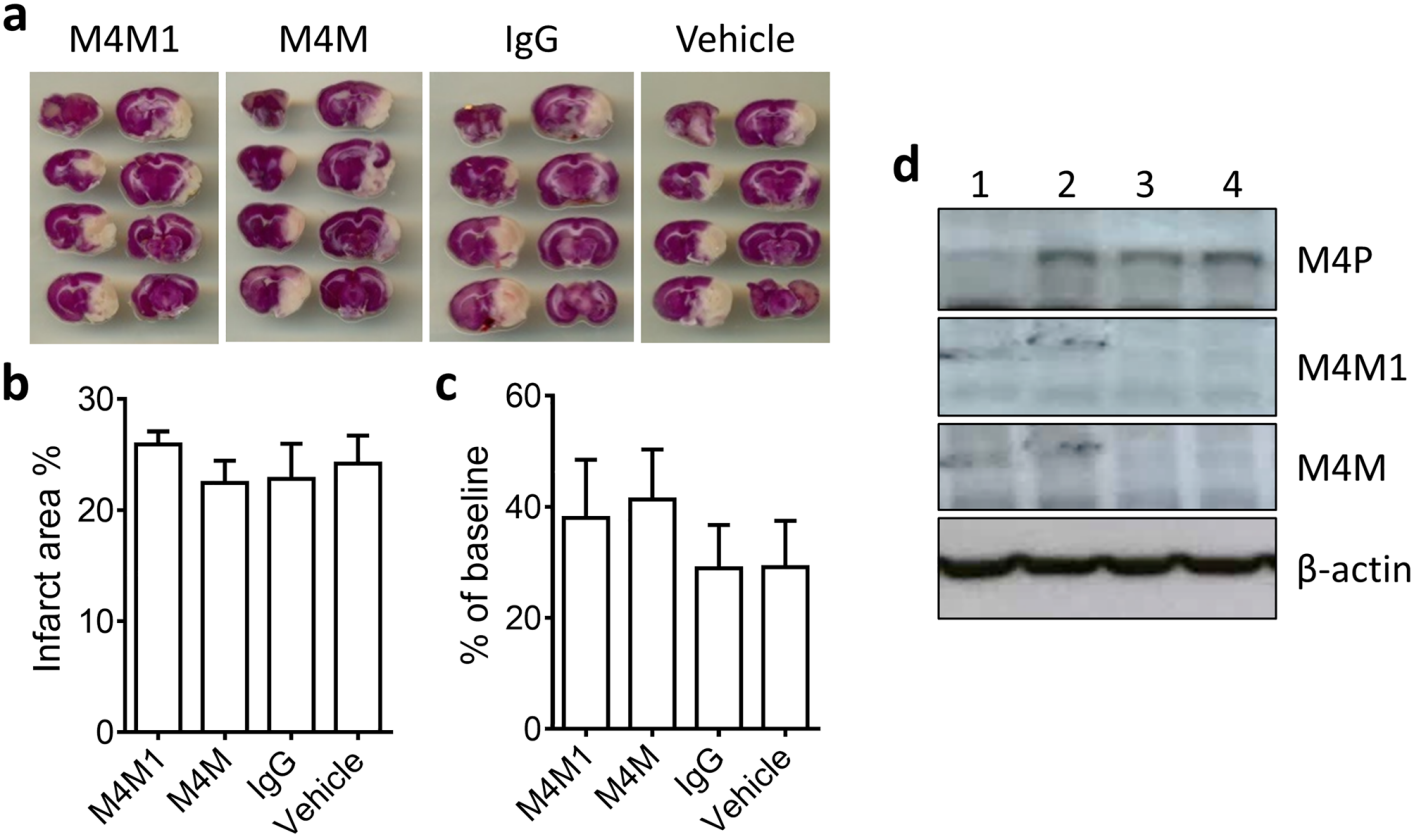
Both M4M and M4M1 failed to improve stroke outcome in wild type rats. (**a**) Transient stroke model was created by occluding left middle cerebral arteries for 3 h. Control mouse IgG, M4M1, M4M (each at 100 μg) and vehicle were injected 1 h before recanalization. TTC staining was performed 1 day after operation. (**b**) Summary of infarct area at 1 day after stroke. (**c**) Assessment of motor functions by Rotarod test 1 day after stroke induction. (**d**) Using western blot to detect TRPM4 expression within the ipsilateral hemispheres 1 day after stroke induction. Lane 1–4: sham operation, control mouse IgG, M4M1 and M4M. Full-length blots are presented in Supplementary Fig. S3. For (**a**–**d**), n = 8 rats per group. No significance was detected in (**b,c**) using one-way ANOVA with Bonferroni post-hoc analysis.

To evaluate the in vivo effect of M4M and M4M1, transient ischemic stroke was performed on wild type Wistar rats. Left middle cerebral artery was occluded for 3 h followed by reperfusion. We used this model to successfully detect the therapeutic potential of M4P^24^. In line with clinical scenario, the antibodies were delivered one hour before recanalization. Based on our previous study on M4P^24^, a dose of 100 µg M4M or M4M1 was selected and injected into the animals via the tail vein. One day after operation, motor functions of the animals were evaluated and the brains were collected. In contrast to the beneficial effect observed in M4P treatment^24^, both M4M1 and M4M demonstrated no difference in terms of infarct volume and motor functions comparing to control IgG or vehicle (Fig. 6a–c). As in vitro studies showed that M4M and M4M1 could only bind to human TRPM4, we employed western blot on tissues harvested from the rat stroke brains. M4P, which was originally designed to target rodent TRPM4, detected an upregulation of TRPM4 following stroke induction. As expected, both M4M and M4M1 failed to identify rodent TRPM4 expression (Fig. 6d). Therefore, it can be concluded that the lack of therapeutic efficacy in M4M and M4M1 is most likely because they are not able to bind to rodent TRPM4.

**Figure 7:**
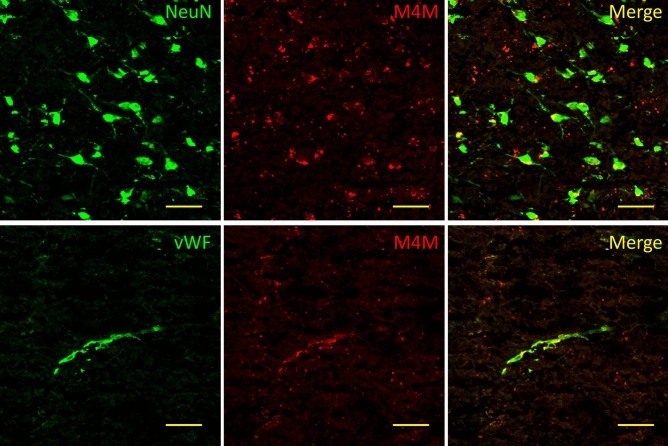
Immunofluorescent staining of human stroke brain tissues. M4M was used to stain human TRPM4 with neuronal marker NeuN and vascular marker vWF. Scale bars: 50 µm.

As M4M has no effect in rodent stroke model, we next examined whether M4M could stain human TRPM4 in stroke brain. Using brain tissues from a hemorrhagic stroke patient, M4M was found partly localized with neuronal marker NeuN and vascular endothelial marker vWF (Fig. 7).

## Discussion

A polyclonal antibody M4P against rat TRPM4 has been generated previously in our lab^24^. M4P could bind TRPM4 from extracellular space and inhibits TRPM4 activity. Therapeutically, intravenous application of M4P was shown to reduce reperfusion injury in a rat stroke model. To translate the research into clinical usage, we aim to generate an antibody against human TRPM4 as the binding sequence in human TRPM4 is different from rodents^24^.

The 28-amino acid polypeptide that was used to generate M4P has 58.3% homology to human TRPM4^24^. After analyzing the hydrophobicity of the human sequence, several amino acids at the C-terminus were excluded and the sequence is reduced to 21 which is now 57.1% homologous to rat TRPM4. Two monoclonal antibodies, M4M1 and M4M, were generated and M4M has demonstrated better blocking effect than M4M1. Electrophysiological studies on HBMECs and HEK 293 cells expressing human TRPM4 indicate that M4M directly blocks TRPM4 currents. In together with immunostaining and surface biotinylation results, we confirm that M4M could suppress TRPM4 functions via two mechanisms: (1) inhibit TRPM4 current in live cells via binding to the channel from extracellular space; (2) downregulate surface TRPM4 expression via forming antibody-channel complex. In therapeutic antibodies for cancer treatment, receptor-antibody complex is known to induce endocytosis and subsequent protein degradation^33^. Comparing to small molecule blockers, this additional mechanism suggests that M4M could yield a stronger blocking effect and the inhibitory effect might last longer.

The therapeutic potential of M4M was examined and verified in cultured HBMECs. Hypoxia is known to increase intracellular Ca^2+^ concentration and reduce ATP level, both of which have been shown to activate TRPM4 channel^5^. Excessive Na^+^ ion influx via TRPM4 leads to cell swelling as water travels together with Na^+^^15^. In cultured HBMECs receiving hypoxia treatment, pre-incubation with M4M successfully blocked TRPM4 currents and inhibited cell swelling. This result indicates that M4M can be used to treat neurological disorders with endothelial damage due to hypoxia such as stroke. However, as M4M could not block rodent TRPM4, no therapeutic effect was observed in a stroke model using wild type rats. A transgenic animal model carrying human TRPM4 sequence is necessary to characterize the in vivo effect of M4M. Nevertheless, the potential effect of M4M on stroke is examined on human stroke tissues. M4M was found partly localized to neurons and vascular endothelium. The differential expression of TRPM4 after stroke has been reported previously in an animal model^26^. It was suggested that the impact of hypoxia/ischemia is heterogeneous among the surviving cells, leading to a differential expression of TRPM4.

In stroke, there are at least two types of cells that can be salvaged by TRPM4 blockade: neuron and vascular endothelial cell^23,24,26^. For vascular endothelial cell, TRPM4 blocking antibody can directly bind to TRPM4 from the lumen of vasculature. However, to act on neuronal TRPM4, TRPM4 blocking antibody needs to enter the brain parenchyma. One major concern about using therapeutic antibody in brain is the presence of blood–brain barrier (BBB). BBB represents a major obstacle for drug delivery to the central nervous system. In healthy brains, large molecules such as antibody are difficult to cross BBB. However, in hypoxic/ischemic diseases such as stroke, BBB integrity is disrupted, allowing antibodies to cross BBB^34^. The ability of M4P to migrate into brain after stroke has been proved in our previous study^24^. In a rat stroke reperfusion model, M4P was shown to enter the brain via a leaky BBB and yielded a therapeutic outcome^24^.

Therapeutic antibodies have been proposed to treat stroke by targeting various key molecules involved in stroke pathophysiology^34^. Although results from animal studies are encouraging, these antibodies failed to improve stroke outcome due to side effects either from the off-target effect or from the immunological effects of the antibody itself. With the technology of antibody humanization, sequence of non-human origin in the antibody can be replaced by human sequence to ameliorate rejection. However, humanized antibodies sometimes still trigger human anti-human responses^35^. Therefore, the challenge in antibody humanization is to balance between the maintenance of biological binding affinity and the reduction of adverse effects.

The potential off-target effect of TRPM4 inhibition is expected to be low. One evidence is that TRPM4-deficient mice are viable and fertile with no obvious anatomical abnormalities^5^, suggesting that blocking TRPM4 can be well tolerated. In our past studies using siRNA or M4P to inhibit TRPM4 in stroke animal models^23,24,26^, we did not encounter any side effect on the functions of vital organs. More importantly, many diseases relating to TRPM4 malfunction is a result of gain-of-function. Under hypoxic conditions, TRPM4 becomes more active as the channel activity is enhanced by higher intracellular Ca^2+^ level with ATP depletion^1^. Therefore, TRPM4 inhibition is more hypoxic specific and can be restricted to the disease affected areas, limiting the side effect on healthy tissues or organs where TRPM4 activity is low. This was proved in our study on HBMECs (Fig. 4d). Under normoxic conditions at 0 min, the baseline currents are similar in non-treated group, M4M group and IgG group. As M4M has demonstrated no effect on normoxic cells, we can postulate that TRPM4 is not a major contributor to the basal currents in HBMECs.

Comparing to TRPM4 blockers 9-phenanthrol and glibenclamide, which can nonspecifically inhibit TMEM16A channel^17^, transient outward, rapid inward rectifier K^+^ channels^18^, and K_ATP_ channels^19^ respectively, TRPM4 blocking antibodies are more specific to the channel. We have shown previously that M4P has no effect on close members of TRPM4 such as TRPM2, TRPM5, and TRPM7^24,27^. The fact that M4P and M4M do not have species cross-reactivity further supports the specificity of TRPM4 blocking antibodies.

In conclusion, we have generated a mouse monoclonal antibody against human TRPM4, and further proved that the function of human TRPM4 channel can be inhibited by this antibody. It is worth noting that M4M is a monoclonal antibody derived from mouse hybridoma cells. The murine origin could generate severe side effects if M4M is used in human directly. Therefore, to achieve a clinical outcome, the antibody must be humanized to minimize the potential effect of rejection.

## Supplementary Information

Supplementary Information.
