# Supplementary material for: Development and characterization of a monoclonal antibody blocking human TRPM4 channel
Source: Sci Rep. 2021 May 17;11:10411. doi: 10.1038/s41598-021-89935-5 (PMC8129085; doi:10.1038/s41598-021-89935-5)

**Development and characterization of a monoclonal antibody blocking human TRPM4 channel**

See Wee Low[1]*, Yahui Gao[1]*, Shunhui Wei[1]*, Bo Chen[1], Bernd Nilius[2], Ping Liao[1,3,4]†

**Supplementary Figure S1**: Full-length blots for western blot on HEK 293 cells transfected with pcDNA c-Myc empty vector (lane 1), mouse TRPM4 (lane 2) and human TRPM4 (lane 3). M4M and M4M1 are two mouse monoclonal antibodies raised against human TRPM4. M4P is a polyclonal antibody raised against rodent TRPM4.

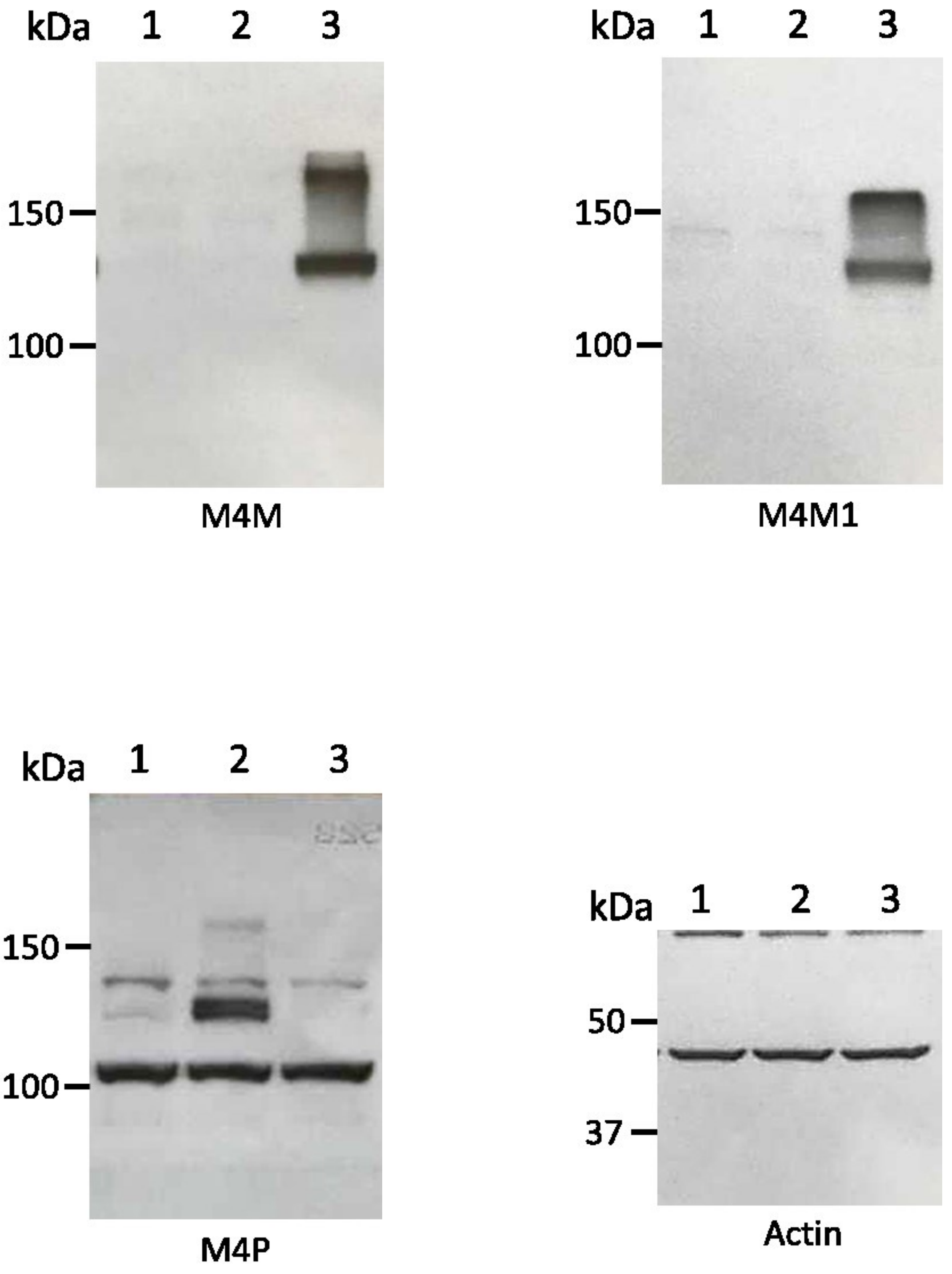

**Supplementary Figure S2**: Full-length blots for western blot shows detection of surface biotinylated human TRPM4 protein.

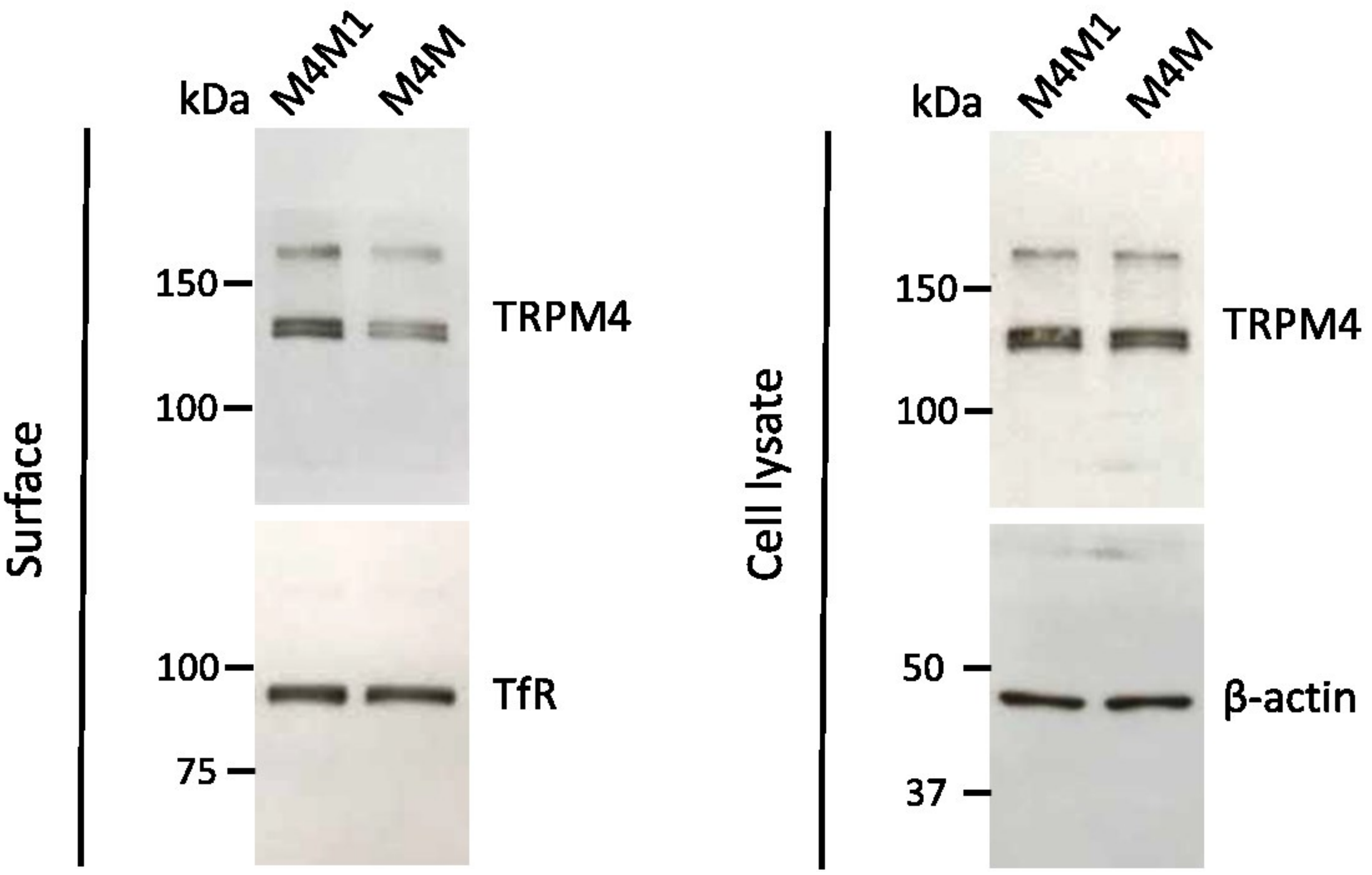

**Supplementary Figure S3**: Full-length blots for western blot shows TRPM4 expression within the ipsilateral hemispheres 1 day after stroke induction. Lane 1-4: sham operation, control mouse IgG, M4M1 and M4M.

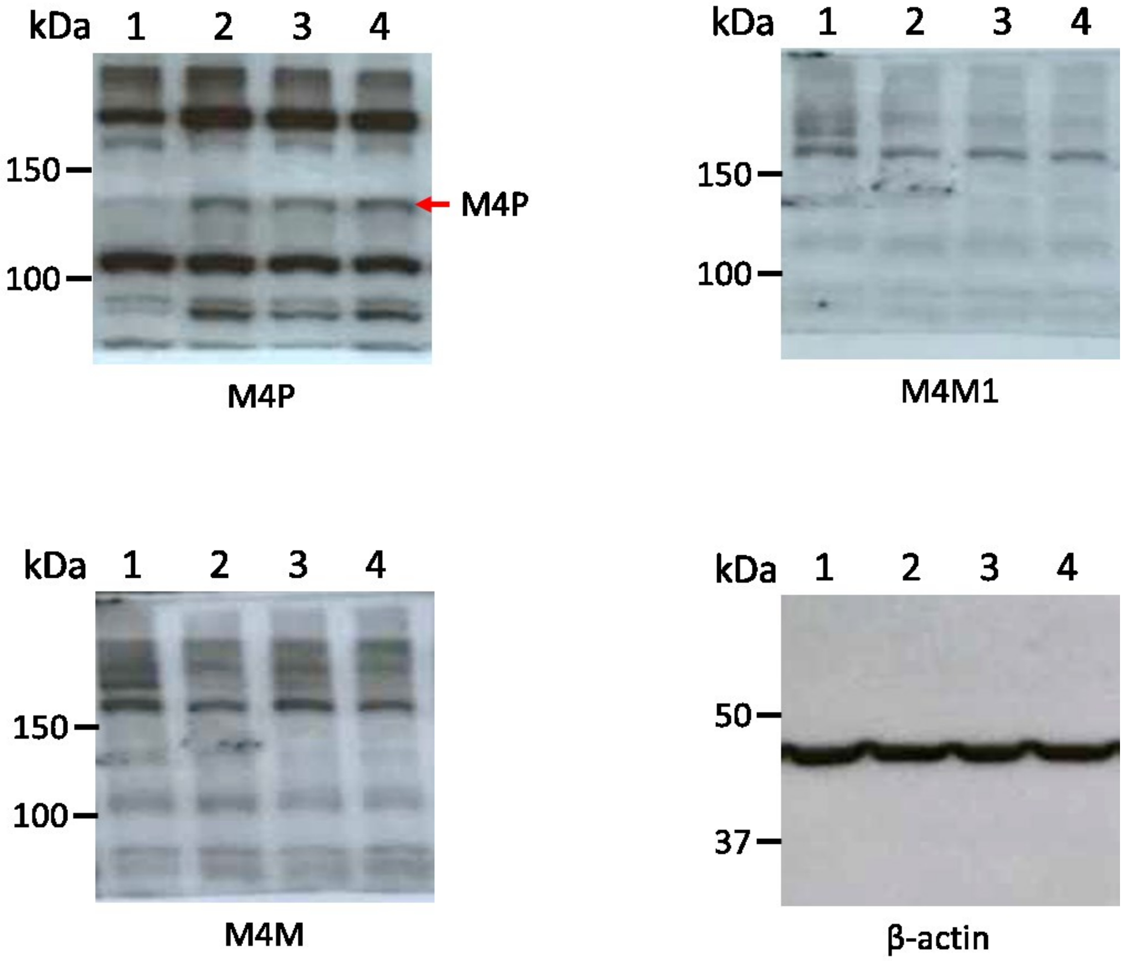

Supplement: Supplementary file 1 — Supplementary Information. [file 41598_2021_89935_MOESM1_ESM.pdf]
